# Synthesis of the oxysterol, 24(*S*), 25-epoxycholesterol, parallels cholesterol production and may protect against cellular accumulation of newly-synthesized cholesterol

**DOI:** 10.1186/1476-511X-6-10

**Published:** 2007-04-05

**Authors:** Jenny Wong, Carmel M Quinn, Andrew J Brown

**Affiliations:** 1 School of Biotechnology and Biomolecular Sciences, The University of New South Wales, Sydney, Australia; 2 Centre for Vascular Research at The University of New South Wales and Department of Haematology, Prince of Wales Hospital, Sydney, Australia

## Abstract

**Aim:**

The effects of 24(*S*),25-epoxycholesterol (24,25EC) on aspects of cholesterol homeostasis is well-documented. When added to cells, 24,25EC decreases cholesterol synthesis and up-regulates cholesterol efflux genes, including ABCA1. Synthesis of 24,25EC occurs in a shunt of the mevalonate pathway which also produces cholesterol. Therefore, 24,25EC synthesis should be subject to the same negative feedback regulation as cholesterol synthesis. To date, no role has been ascribed to 24,25EC in light of the fact that increased accumulation of cholesterol should decrease formation of this oxysterol through feedback inhibition. This leads to the intriguing paradox: why inhibit production of an apparently important regulator of cholesterol homeostasis when it is needed most?

**Methods:**

We used a combination of pharmacological and genetic approaches in Chinese Hamster Ovary cell-lines to investigate this paradox. Endogenous synthesis of 24,25EC was manipulated using partial inhibition of the enzyme, Oxidosqualene Cyclase. Changes in cholesterol and 24,25EC synthesis were determined using metabolic labelling with [1-^14^C]-acetate, thin-layer chromatography and phosphorimaging. Transcriptional effects mediated via SREBP and LXR were analysed by luciferase reporter assays.

**Results:**

We showed that cholesterol addition to cells lead to a rapid and preferential inhibition of 24,25EC synthesis. Addition of 24,25EC resulted in parallel inhibition of 24,25EC and cholesterol synthesis. Furthermore, we used a variety of approaches to examine the relationship between cholesterol and 24,25EC synthesis, including cell-lines with different rates of cholesterol synthesis, varying cholesterol synthetic rates by pre-treatment with a statin, or lipoprotein cholesterol loading of macrophages. In all cases, we showed that 24,25EC synthesis faithfully tracked cholesterol synthesis. Moreover, changes in 24,25EC synthesis exerted downstream effects, reducing SREBP transcriptional activity whilst increasing ABCA1 and LXR transcriptional activity.

**Conclusion:**

Our results show that 24,25EC synthesis parallels cholesterol synthesis, consistent with this oxysterol functioning as a safety valve to protect against the accumulation of newly-synthesised cholesterol (as opposed to exogenously-derived cholesterol). Considering that 24,25EC is capable of being produced in all cholesterogenic cells, we propose that production of 24,25EC may represent a ubiquitous defence mechanism.

## Background

Oxygenated cholesterol derivatives or oxysterols are potential regulatory molecules in cholesterol homeostasis. The regulatory effect of oxysterols was first identified by Kandutsch and colleagues [1] who showed that the suppressive effect of cholesterol on its own synthesis may be mediated by endogenously-produced oxysterols. This became known as the "Oxysterol Hypothesis of Cholesterol Homeostasis" [1].

Certain oxysterols can act as suppressors of 3-hydroxy-3-methylglutaryl (HMG)-CoA reductase, the rate-limiting enzyme in the mevalonate pathway which also synthesises cholesterol [2]. It has been demonstrated that oxysterols decrease the expression of cholesterol synthetic and uptake genes by suppressing sterol regulatory element-binding protein-2 (SREBP-2) mediated transcription [3,4]. Moreover, certain oxysterols can act as ligands for the Liver × Receptor (LXR), which mediates transcription of cholesterol export genes, such as the ATP-binding cassette transporter, ABCA1 [5-7]. Most of the common physiological oxysterols characterised (such as the hydroxycholesterols) are derived from enzymatic oxidation of cholesterol, either from circulating lipoproteins or from synthesised cholesterol [8,9].

The oxysterol 24(*S*),25-epoxycholesterol (24,25EC) is unique in that it is not derived from cholesterol. Instead, 24,25EC is produced *de novo *in the mevalonate pathway which simultaneously synthesizes cholesterol. 24,25EC was first characterised by Nelson, Steckbeck and Spencer [10] who showed that 24,25EC was derived from acetyl CoA via a shunt in the mevalonate pathway. Since then, exogenous addition of 24,25EC has been shown *in vitro *to decrease HMG-CoA reductase activity [11], to suppress SREBP processing [4], and to potently activate LXR-gene targets [6,7,12]. We have previously shown that statins inhibit the production of 24,25EC in human macrophages under basal conditions leading to decreases in LXR target gene expression including ABCA1 and ABCG1 [13]. Moreover, beneficial effects on cholesterol homeostasis have been reported *in vitro *[13-17] and *in vivo *[18-21] when endogenous synthesis of 24,25EC is stimulated. Two recent reviews [22,23], have highlighted the interest in 24,25EC as a central regulator of cholesterol homeostasis [22].

Feedback regulation of cholesterol synthesis is well established [2]. When cholesterol levels in the cell are high, SREBP-processing is inhibited resulting in down-regulation of SREBP-target genes, including HMG-CoA reductase [2]. Considering that cholesterol and 24,25EC synthesis utilize the same enzymes and cofactors, synthesis of 24,25EC would be expected to decrease when cellular cholesterol accumulates and feeds back on the mevalonate pathway. This leads to the intriguing question: why inhibit production of a compound that potently regulates cholesterol homeostasis when it is needed most? To date, no publication on 24,25EC has addressed or even raised this important issue. Our current work helps to resolve this paradox by showing that although cholesterol addition potently inhibits 24,25EC synthesis, synthesis of this oxysterol tends to track cholesterol synthesis under a variety circumstances. This has lead us to propose that production of this oxysterol represents a ubiquitous defence mechanism against cellular accumulation of cholesterol that is newly-synthesised in the mevalonate pathway (as opposed to exogenously-derived cholesterol).

## Results

### Cholesterol accumulation inhibits 24(*S*),25-epoxycholesterol synthesis

**Figure 1:**
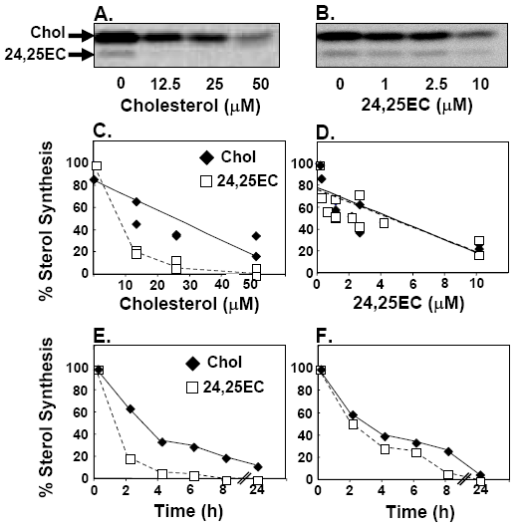
**Exogenously-added sterols feed back on cholesterol and 24,25EC synthesis**. CHO-7 cells were incubated with either increasing concentrations (**A**) or 50 μM (**E**) of cholesterol complexed to methyl-β-cyclodextrin; or either increasing concentrations (**B**) or 10 μM (**F**) of 24,25EC in the presence of [1-^14^C]-acetate for 24 h or (**E, F**) labelled 2 h before the indicated time of harvest. Neutral lipid extracts were separated by thin-layer chromatography and bands corresponding to authentic cholesterol and 24,25EC were visualized by phosphorimager. These phosphorimages are representative of 2 separate experiments. Bands corresponding to cholesterol and 24,25EC were quantified by densitometry. For (**C, D**) data sets from 2 (C) or 3 (D) separate experiments represented by Figure 1A and Figure 1B were pooled. Equation for the lines of best fit (**C**) cholesterol (solid diamond; solid line) y = -1.35x + 84.79; R^2 ^= 0.75; P < 0.01; (**D**) 24,25EC (empty square; dashed line) y = -5.68x + 76; R^2 ^= 0.58; P < 0.01; cholesterol (solid diamond; solid line) y = -5.96x + 78; R^2^= 0.63; P < 0.01. For (**E, F**) data sets are representativeof n = 2 separate experiments.

Considering that both 24,25EC and cholesterol are synthesised in the mevalonate pathway, we would predict that 24,25EC synthesis should also be decreased upon cellular cholesterol accumulation as a consequence of feedback inhibition of the mevalonate pathway. To test this, we utilised Chinese Hamster Ovary (CHO) cells, a cell-line commonly-used to study cholesterol homeostasis that has been instrumental in the identification of many of the major components of the cholesterol homeostatic machinery [24]. CHO-7 cells were cholesterol-loaded by incubation with incremental concentrations of cholesterol complexed to methyl-β-cyclodextrin in the presence of [1-^14^C]-acetate. Non-saponifiable lipids were extracted, separated by thin layer chromatography and phosphorimaged. Quantification of cholesterol and 24,25EC by densitometry of the phosphorimage or by scintillation counting yielded linear responses in the standard curve for both sterols (Additional file 1). Thus, changes in cholesterol and 24,25EC synthesis were subsequently quantified by densitometry of the phosphorimage. Cholesterol-loading decreased synthesis of both sterols as predicted. However, 24,25EC synthesis appeared to decrease preferentially (Fig. 1A, C). Incubation of CHO-7 cells with 24,25EC was more potent in inhibiting sterol synthesis than cholesterol-loading (Fig. 1D*vs*. 1C) and decreased both cholesterol and 24,25EC synthesis to a similar extent (Fig. 1B, D).

The effects of added cholesterol or 24,25EC on sterol synthesis were then examined over time (Fig. 1E, F). Cholesterol-loading and 24,25EC incubation decreased the synthesis of the two sterols within hours of treatment. The preferential decrease in 24,25EC synthesis in response to cholesterol-loading was evident within the first two hours (Fig. 1E).

Therefore, 24,25EC synthesis is inhibited in response to added sterols. We postulate that 24,25EC may be functioning to protect against intracellular accumulation of cholesterol when its synthesis in the mevalonate pathway is active.

### Synthesis of 24(*S*),25-epoxycholesterol faithfully tracks cholesterol synthesis

**Figure 2:**
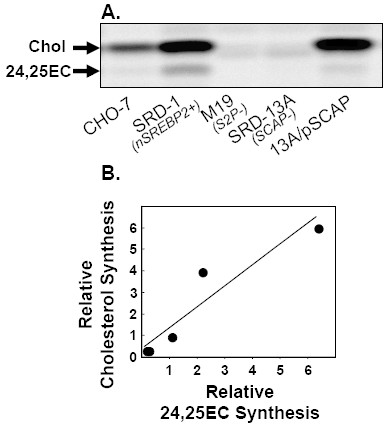
**Endogenously-produced 24,25EC correlates with cholesterol synthesis in different CHO cell-lines**. (**A**) Various Chinese Hamster Ovary cell-types with different abilities to synthesise cholesterol were incubated in the presence of [1-^14^C]-acetate for 24 h. KEY: 1. CHO-7; 2. SRD-1; 3. M19; 4. SRD-13A; 5. 13A/pSCAP. For more details about these cell-lines, see [25]. Neutral lipid extracts were separated by thin-layer chromatography and bands corresponding to authentic cholesterol and 24,25EC were visualized by phosphorimager and quantified by densitometry. Equations for the lines of best fit (**B**) y = 0.97x + 0.39; R^2 ^= 0.89; P < 0.03. Values are means from 3 separate experiments set relative to CHO-7 cells which was assigned an arbitrary value of 1.

If 24,25EC protects against newly-synthesised cholesterol, we would predict that syntheses of these two sterols should correlate. This was observed when addition of 24,25EC decreased synthesis of 24,25EC and cholesterol in parallel (Fig. 1D, F). We investigated this further using a variety of Chinese Hamster Ovary cell-lines containing specific mutations in the SREBP-processing pathway which lead to altered cholesterol biosynthesis rates (details presented in [25]). Regardless of the mutation, synthesis of 24,25EC was correlated with cholesterol synthesis (Fig. 2A, B).

**Figure 3:**
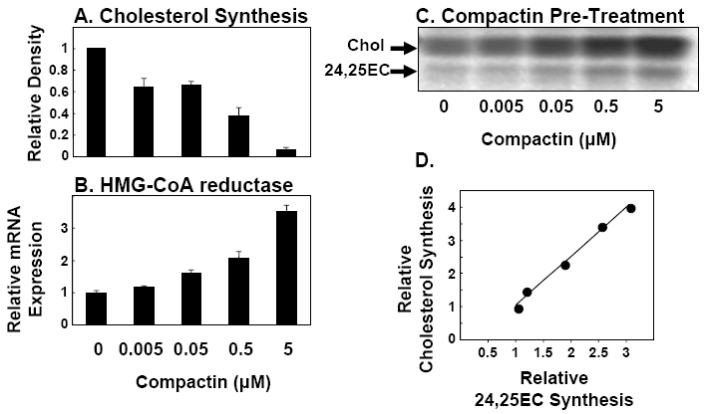
**Endogenously-produced 24,25EC correlates with cholesterol synthesis when cholesterol synthesis is stimulated by statin-pre-treatment**. (**A**) CHO-7 cells were incubated with the indicated concentrations of compactin in the presence of [1-^14^C]-acetate for 24 h. Neutral lipid extracts were separated by thin-layer chromatography and bands corresponding to authentic cholesterol and 24,25EC were visualized by phosphorimager and quantified by densitometry. Values are means+SEM from 3 separate experiments. (**B, C**) CHO-7 cells were pre-incubated with the indicated concentrations of compactin for 24 h. (**B**) mRNA levels for HMG-CoA reductase was measured using QRT-PCR. Data are presented relative to vehicle-treated controls and are means+SEM (3 replicate cultures representative of 2 separate experiments). (**C**) Cells were then incubated in the presence of [1-^14^C]-acetate for 2 h. Neutral lipid extracts were separated by thin-layer chromatography and bands corresponding to authentic cholesterol and 24,25EC were visualized by phosphorimager and quantified by densitometry. Equations for the lines of best fit (**D**) y = 1.47x - 0.42; R^2 ^= 0.99; P < 0.001. Values are means from 2 separate experiments set relative to the vehicle-treated control condition which was assigned an arbitrary value of 1.

In another experimental approach, the rate of cholesterol synthesis was varied in CHO-7 cells by pre-incubating with increasing concentrations of the archetypal statin, compactin. Statins decrease cholesterol synthesis by inhibiting HMG-CoA reductase (Fig. 3A). The resulting cholesterol depletion induces SREBP activation [26] and consequently up-regulates expression of key rate-limiting cholesterol biosynthetic enzymes, including HMG-CoA reductase [13,25] (Fig. 3B). Hence, after pre-incubation when compactin is removed, there is an increase in the rate of cholesterol synthesis in the mevalonate pathway (Fig. 3C). The induction in cholesterol synthesis increased by up to four-fold and yet again was closely tracked by 24,25EC synthesis (Fig. 3D).

**Figure 4:**
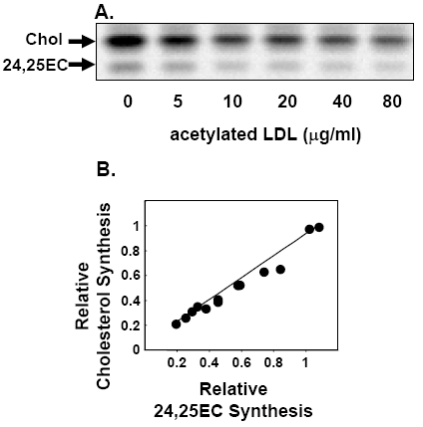
**Endogenously-produced 24,25EC correlates with cholesterol synthesis when macrophages are cholesterol-loaded**. (**A**) THP-1 monocytes differentiated into macrophages for 3 d in 50 ng/μl of PMA. THP-1 macrophages were loaded with acetylated LDL for 24 h whilst labelling with [1-^14^C]-acetate. Neutral lipid extracts were separated by thin-layer chromatography and bands corresponding to authentic cholesterol and 24,25EC were visualized by phosphorimager and quantified by densitometry. Neutral lipid extracts were separated by thin-layer chromatography and bands corresponding to authentic cholesterol and 24,25EC were visualized by phosphorimager and quantified by densitometry. Equations for the lines of best fit (**B**) y = 0.88x; R^2 ^= 0.97; P < 0.0001. Values are pooled from 2 separate experiments set relative to the vehicle-treated control condition which was assigned an arbitrary value of 1.

In our final approach, we studied the effects of lipoprotein cholesterol loading on cholesterol and 24,25EC synthesis in macrophages, a cell-type particularly relevant to the etiology of atherosclerosis. For these experiments, we utilized the human macrophage-like cell-line, THP-1 [13]. Cholesterol loading of THP-1 human macrophages with acetylated LDL decreased both cholesterol and 24,25EC synthesis in unison (Fig. 4A, B).

Taken together, these positive correlations between cholesterol and 24,25EC synthesis and the potent suppressive effect of added 24,25EC on cholesterol synthesis (Fig. 1B, 1D) support its role as a feedback regulator when cholesterol is being actively synthesised.

### 24(*S*),25-epoxycholesterol regulates SREBP- and LXR-mediated transcription

**Figure 5:**
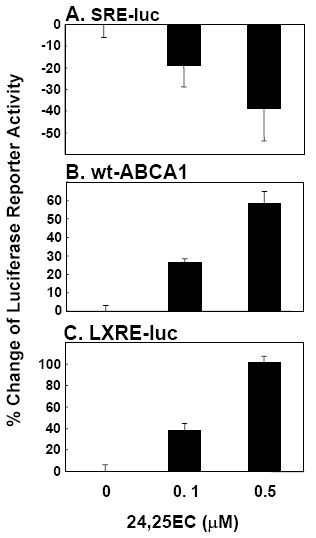
**Added 24(*S*),25-epoxycholesterol regulates SREBP- and LXR-mediated transcription**. (**A-C**) CHO-7 cells were transiently transfected for 24 h with phRL-TK Renilla internal control plasmid together with either (**A**) SRE-luc, (**B**) pGL3-hABCA1 wild-type, (**C**) LXRE-luc. Following transfection, cells were incubated for 24 h in the absence or presence of indicated concentrations of 24,25EC. Data are presented as percentage change relative to the vehicle-treated control condition. (**A**,**B**) Values are mean+SEM (n = 3 separate experiments). (**C**) Values aremean+SEM (n = 3 replicate cultures representative of 3 separate experiments).

Addition of certain oxysterols including 24,25EC, are known to block SREBP-2 activation in the cell [3,4]. To assess changes in gene transcription, we employed luciferase reporter constructs containing tandem repeats of the motif at which transcriptional changes take place. The effect of added 24,25EC on SREBP-mediated transcription was examined using a luciferase reporter construct containing 6 tandem copies of the classical sterol response element from the promoter of the LDL receptor, ATCACCCCAC (SRE-luc) [27,28]. Hence, changes in SRE-luc reporter activity reflect SREBP-mediated transcription. 24,25EC is a potent activator of LXR-target genes [6,12] including the cholesterol efflux gene, ABCA1 [29,30]. To assess LXR-mediated gene transcription, we used a luciferase reporter construct containing a 1 kb fragment of the human ABCA1 promoter [30] as well as a luciferase reporter containing 3 repeats of the minimal DR4 motif (LXRE-luc). In agreement with our previous findings [25], addition of increasing concentrations of 24,25EC lead to a decrease in SRE reporter activity and to reciprocal increases in ABCA1 and LXRE-luc reporter activity (Fig. 5A–C)).

**Figure 6:**
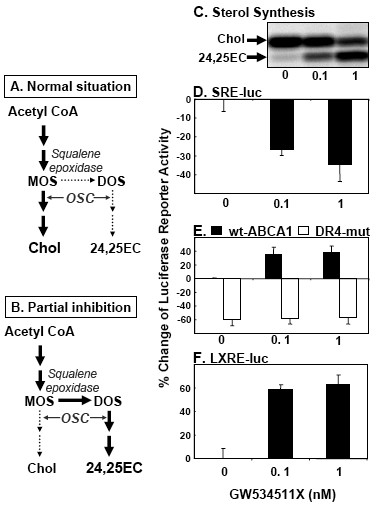
**Endogenous 24(*S*),25-epoxycholesterol regulates SREBP- and LXR-mediated transcription**. **(A) **Under normal conditions, cholesterol synthesis is favoured but some 24,25EC is still produced. **(B) **Since oxidosqualene cyclase (OSC) preferentially cyclises 2,3(*S*):22(*S*),23-dioxidosqualene (DOS) over 2,3(*S*)-monooxidosqualene (MOS), partial inhibition of OSC favours synthesis of 24,25EC. (**C**) CHO-7 cells were incubated with increasing concentrations of GW534511X in the presence of [1-^14^C]-acetate for 24 h. Neutral lipid extracts were separated by thin-layer chromatography and bands corresponding to authentic cholesterol and 24,25EC were visualized by phosphorimager. This phosphorimage is representative of 3 separate experiments. (**D-F**) CHO-7 cells were transiently transfected for 24 h with phRL-TK Renilla internal control plasmid together with either (**D**) SRE-luc, (**E**) pGL3-hABCA1 wild-type or DR4 mutant, (**F**) LXRE-luc. Following transfection, cells were incubated for 24 h in the absence or presence of indicated concentrations of GW534511X. Data are presented as percentage change relative to (**E**) the pGL3-hABCA1 wild-type construct vehicle-treated control condition or (**D**, **F**) vehicle-treated control condition. (**D, E, F**) are means+SEM(n = 3 separate experiments); for (**E**) DR4 mutant are means+halfrange (n = 2 separate experiments).

It is well documented that addition of 24,25EC has marked effects on parameters associated with cholesterol homeostasis [6,13,14,25]. We aimed to determine if endogenous 24,25EC also has the expected effects on SREBP- and LXR- mediated transcription. A specific inhibitor of the enzyme 2,3-oxidosqualene cyclase (OSC), GW534511X, was used to partially inhibit the activity of this enzyme, hence increasing the amount of 24,25EC produced in the shunt pathway (illustrated in Fig. 6A, B). We and others have shown that use of high OSC inhibitor concentrations increase production of 2,3(*S*):22(*S*),23-dioxidosqualene (DOS) and almost completely abolish cholesterol synthesis [13,14,17]. Since these may produce confounding effects, lower inhibitor concentrations were used. In agreement with previous studies [13-16,18,20], we found that partial OSC inhibition increased 24,25EC synthesis and decreased cholesterol synthesis (Fig. 6C).

When endogenous 24,25EC synthesis was increased by partial OSC inhibition (Fig. 6C), SREBP-reporter activity was decreased (Fig. 6D). In addition, we assessed SREBP-processing through use of an SREBP-reporter fusion construct [26] which confirmed that endogenously-produced 24,25EC decreased SREBP-processing (data not shown).

ABCA1 luciferase reporter activity increased in accordance with the increase in 24,25EC synthesis when OSC was partially inhibited by 0.1 nM and 1 nM GW534511X (Fig. 6E). To confirm that ABCA1 promoter activity was indeed the result of increased endogenous synthesis of LXR ligand 24,25EC, two approaches were employed. Firstly, the wild-type ABCA1 reporter construct was mutated at the LXR response element, DR4 motif (DR4 mutant). Reporter activity of the DR4 mutant construct was lower than that of the wild-type construct and displayed no response to changes in endogenous 24,25EC synthesis (Fig. 6E). Secondly, we observed similar effects of GW534511X treatment on LXRE-luc activity as compared with ABCA1 reporter activity (Fig. 6F). This confirms that the changes induced by GW534511X were mediated through the LXR response element and are most likely due to endogenous 24,25EC synthesis. This result also suggests that the effect of endogenously-produced 24,25EC should be applicable to other LXR-target genes.

Collectively, our results support the idea that endogenously-produced 24,25EC may act as a feedback regulator to protect the cell against the cholesterol accumulation of newly-synthesized cholesterol.

## Discussion

24,25EC, produced *de novo *in a shunt of the mevalonate pathway, is a promising physiological oxysterol mediator of cholesterol homeostasis [22,23]. The shunt pathway runs parallel to cholesterol synthesis. Thus enzymes for the synthesis of both cholesterol and 24,25EC are shared and subject to the same regulation. Paradoxically, synthesis of 24,25EC decreases when cholesterol accumulates and feeds back, preventing activity of the mevalonate pathway i.e. when cellular cholesterol is high and the need for 24,25EC should be greatest. Our current work helps to clarify this paradox by proposing a previously unrecognized role of 24,25EC as a defence mechanism against cellular accumulation of newly-synthesized cholesterol as opposed to exogenously-derived cholesterol from lipoproteins. Below we present several lines of evidence in support of this contention.

Firstly, we showed that addition of pure cholesterol to cells preferentially shut down synthesis of 24,25EC (Fig. 1C, E). Moreover, lipoprotein-cholesterol loading of macrophages also decreased 24,25EC synthesis (Fig. 4A). These results confirm our prediction that 24,25EC's role can not be to protect against accumulation of exogenous cholesterol and suggests a feed-back system designed to preferentially attenuate levels of this oxysterol when cholesterol synthesis is not required.

We further explored the role of 24,25EC synthesis as a feed-back mechanism by employing four different experimental approaches (Fig. 1B, Fig. 2B, 3D, 4B). We found that synthesis of 24,25EC faithfully tracks cholesterol synthesis in each case. Firstly, we suppressed the activity of the mevalonate pathway by addition of exogenous 24,25EC. Both cholesterol and 24,25EC synthesis decreased in unison and the effects observed were dependent on concentration and time (Fig. 1D, F). Secondly, through the use of mutant CHO cell-lines which exhibit varying rates of cholesterol synthesis, we found that 24,25EC synthesis follows cholesterol synthesis (Fig. 2B). Thirdly, when the biosynthetic activity of the mevalonate pathway was increased by statin-pre-treatment, we also observed parallel increases in cholesterol and 24,25EC synthesis (Fig. 3D). Finally, cholesterol-loading of the human macrophage cell-line, THP-1, produced parallel decreases in cholesterol and 24,25EC synthesis (Fig. 4B).

We also showed that synthesised 24,25EC had the expected downstream effects on LXR- and SREBP-mediated gene transcription. Induction of endogenous 24,25EC synthesis with the OSC inhibitor GW534511X activated ABCA1 transcription (Fig. 6E) in agreement with previous studies which have reported increased ABCA1 mRNA levels in response to partial inhibition of OSC [13,14,17]. The reciprocal effects were observed for SREBP-mediated gene transcription (Fig. 6D) which decreased as 24,25EC synthesis increased. Again, this is in keeping with previous studies [4,14]. The magnitude of effects observed in these luciferase reporter assays is similar to those observed in response to sub-micromolar concentrations of added 24,25EC (Fig. 5).

**Figure 7:**
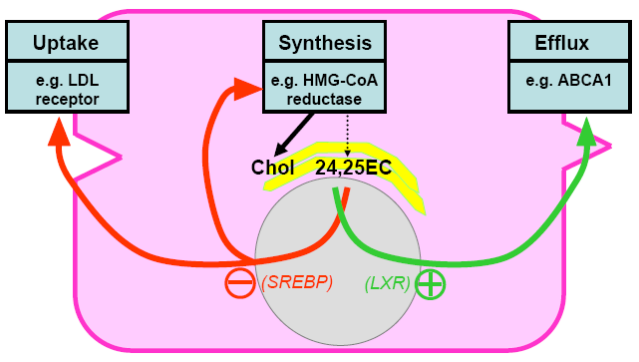
**A working hypothesis showing 24,25EC's role in protecting against accumulation of newly-synthesised cholesterol**. Synthesis of cholesterol is accompanied by synthesis of 24,25EC in the same pathway. 24,25EC can inhibit cholesterol uptake and synthesis by suppressing SREBP activation. 24,25EC can also promote cholesterol efflux by serving as a ligand for LXR target genes, including ABCA1.

Together, the parallel relationships observed between 24,25EC and cholesterol synthesis as well as the downsteam effects of increased 24,25EC synthesis can be rationalized if 24,25EC represents 'a safety-valve' for protecting against the accumulation of newly-synthesized cholesterol (Fig. 7). Where precursors of cholesterol synthesis diverge to form 24,25EC, this oxysterol can then be utilised as a regulatory molecule to decrease cholesterol synthesis, inhibit SREBP-2 mediated transcription, and to upregulate cholesterol efflux processes.

As both 24,25EC and cholesterol are synthesised in the mevalonate pathway, any cell that can synthesize cholesterol also has the capacity to generate 24,25EC. So far, several cell-types have been shown to generate 24,25EC (including hepatocytes [15,17] (Additional file 2), macrophages [13,14] (Fig. 4A), fibroblasts and lung cells [31]). Spencer et al. [32] previously estimated that the human liver contains 10–30 μM of 24,25EC. At this concentration range the activity HMG-CoA reductase could be suppressed by 50% in fibroblast cell culture [32].

The evidence presented herein is, of necessity, correlational. The use of genetic manipulation to test the idea that 24,25EC may represent a ubiquitous defence mechanism against newly-synthesized cholesterol is problematic, since synthesis of cholesterol and 24,25EC occur concurrently using the same enzymes. However, some *in vivo *evidence for this hypothesis was provided by Zhang *et al*. [19], who found that 24,25EC was relatively enriched in the nuclear fraction of rat liver homogenates, placing it in the correct location to act as an *in vivo *LXR ligand. Furthermore, 1 h after administering a bolus of mevalonic acid to rats, hepatic levels of 24,25EC doubled, in contrast to the other oxysterols measured. These observations are consistent with 24,25EC being produced in response to an overactive mevalonate pathway and supports the hypothesis that it can protect the cell from accumulation of newly-synthesized cholesterol.

Previously, we speculated that human macrophages may produce ligands for LXR, depending on the supply of exogenous cholesterol versus the activity of the shunt pathway [13]. When cholesterol status is sufficient, feed-back inhibition of the mevalonate pathway occurs and enzymatic conversion of cholesterol to oxysterols may be the dominant mechanism for up-regulating cholesterol efflux genes like ABCA1, overriding any effects of 24,25EC. Indeed, when THP-1 macrophages were loaded with acetylated LDL, we observed corresponding decreases in cholesterol and 24,25EC synthesis (Fig. 4A). However, the conversion of cholesterol to oxysterols will be cell-type specific, for example, another LXR ligand 24(*S*)-hydroxycholesterol, is synthesized exclusively in the brain and thus probably does not function as a regulatory molecule in other cell-types [8]. In cells capable of synthesizing oxysterols from cholesterol, we propose that 24,25EC may serve as an auxiliary mechanism for when cholesterol synthesis is high. In cell-types unable to convert cholesterol into oxysterol ligands, we propose that 24,25EC may play a greater role in regulating cholesterol homeostasis.

In summary, this is the first study to rationalize the production of 24,25EC as a ubiquitous defence mechanism against newly-synthesized cholesterol. Given the role of 24,25EC in cholesterol homeostasis and the importance of OSC in determining its endogenous production, it is not surprising that this enzyme is of considerable interest as a pharmacological target [14,17,22,23]. Funk and Landes [33] reported that treatment of hamsters and dogs with OSC inhibitors produced various histopathological lesions, including early-stage cataracts. However, these authors concluded that all adverse effects described were exaggerated at the high dosage levels employed and suggested that use of lower doses of hepatosensitive OSC inhibitors should reduce the probability of adverse side-effects in humans [33]. Instead of blocking the entire mevalonate pathway with statins, the idea of manipulating a pre-existing and self-regulating pathway for the production of a physiological oxysterol mediator, that can augment cholesterol removal and decrease uptake and synthesis, is attractive and warrants further evaluation.

## Methods

### Materials

Chemicals and reagents used are listed below with the supplier. From GE Healthcare (formerly Amersham Biosciences): [1-^14^C]-acetic acid, sodium salt (specific activity: 56 mCi/mmol). From Invitrogen: Dulbecco's modified Eagle's medium/Ham's F-12 medium (50/50 mixture); RPMI 1640; L-glutamine; Lipofectamine 2000; new born calf serum; fetal calf serum; penicillin/streptomycin. From Promega: phRL-TK Renilla plasmid. From Sigma: compactin (also called mevastatin); phorbol 12-myristate 13-acetate (PMA); TriReagent; oligonucleotides were synthesized by Sigma-Genosys. From Steraloids: cholesterol; 24(*S*),25-epoxycholesterol. Other reagents were: analytical- or HPLC-grade solvents (EM Science); UltimaGold scintillation fluid (Packard Bioscience). Lipoprotein-deficient serum (LPDS) was prepared from new born calf serum or human serum [34]. Acetylation of low density lipoprotein (LDL) to acetylated LDL was prepared as previously described [35]. Chinese Hamster Ovary cell-lines, were generously provided by Drs. Michael S. Brown and Joseph L. Goldstein (UT Southwestern, Dallas). A reporter construct (pGL3-hABCA1) containing a fragment of the human ABCA1 promoter (-928 bp to +101 bp) was a kind gift from Dr Alan Tall (Columbia University, New York). Another reporter construct (pGL3-TK) was donated by Dr Malcolm Lyons (Western Australian Institute of Medical Research). The 2,3-oxidosqualene cyclase (OSC) inhibitor, GW534511X, was kindly provided by Glaxo Smith-Kline.

### Cell Culture

All cell types used were grown at 37°C in a 5% CO_2 _atmosphere. CHO-7 cells were cultured in Dulbecco's modified Eagle's medium/Ham's F-12 medium (1:1 mixture). These and other Chinese Hamster Ovary cell-lines were maintained as described [25]. THP-1 human monocytes were cultured in RPMI 1640 plus 10% (v/v) FCS supplemented with penicillin/streptomycin 100 U/100 μg/ml and L-glutamine (2 mM). Differentiation into macrophages was achieved by addition of PMA (50 ng/μl) for three days. Treatments were conducted for 24 h in media containing 5% (v/v) LPDS. All treatments were added to cells either in absolute ethanol, dimethyl sulfoxide or water and compared with vehicle-only controls. No cell-toxicity was observed for any treatment at the concentrations employed.

### Cholesterol and 24(*S*),25-Epoxycholesterol synthesis assay

Cells were metabolically labelled with [1-^14^C]-acetic acid for 2 or 24 h during treatments as previously described [13]. THP-1 macrophages were loaded with acetylated LDL whilst labelling with [1-^14^C]-acetic acid for 24 h. Cells were harvested and cell protein was determined by the bicinchoninic acid assay (Pierce). Samples were saponified and standardised for equal protein loading (~150 μg protein) and neutral lipid extracts were separated by thin-layer chromatography. Bands corresponding to authentic cholesterol and 24,25EC were visualized using the FLA-5100 phosphoimager (Fujifilm, Tokyo, Japan) (18–72 h exposure). Quantification of bands by densitometry was conducted with Sciencelab ImageGauge 4.0 Software (Fujifilm). Positive identification of the band corresponding to 24,25EC has previously been performed chemically and by mass spectrometry (see supplemental data of reference [13]). To validate our results based on densitometry of the phosphorimage, we also performed scintillation counting for an experiment that established linearity of responses (Additional file 1). We show that increasing levels of [^14^C]-cholesterol and [^14^C]-24,25EC give proportional increases in response using both methods of quantification. Following thin layer chromatography, bands corresponding to authentic cholesterol and 24,25EC were cut and eluted with methanol:peroxide free diethyl ether (1:1) v/v. Radioactivity was determined by scintillation counting (Packard Tri-Carb 2100 TR Liquid Scintillation Analyzer) using UltimaGold scintillation fluid (5 ml).

### Plasmid constructs

Mutations in the LXR response element (DR4 motif) in a fragment of the hABCA1 promoter that is linked to the firefly luciferase reporter gene [30] was generated using the Quickchange site-directed mutagenesis kit (Stratagene) [25] and sequences were verified. The 6 × SRE-luciferase reporter has been previously described [25]. A 3 × LXRE-luciferase reporter (LXRE-luc) was constructed using a similar approach [25]. Briefly, the 3 × LXRE-luc insert was constructed by annealing complementary sequences of a 3 × repeat containing the DR4 motif (underlined) "tgaatgaccagcagtaacctcagc" [36]; and *Kpn*I and *Xho*I restriction sites synthesized at the 5' and 3' ends respectively (Sigma-Genosys). The annealed insert and a pGL3-TK vector were then sequentially digested with their respective restriction enzymes and both digested insert and vector were then ligated with T4 DNA ligase and transformed into the DH5α *E. coli *strain. Positive clones were verified by sequencing.

### Human ABCA1 promoter activity assay

Promoter analysis was performed as previously described [25]. Briefly, reporter plasmids (250 ng/well) were transfected for 24 h into cells using Lipofectamine 2000 (1 μl/well). The phRL-TK Renilla internal control plasmid (25 ng/well) was co-transfected for normalization of transfection efficiency. After treatment (24 h), cells were washed and resuspended in 100 μl of 1 × passive lysis buffer (Promega). Luciferase assays were performed using the Dual Luciferase Assay Reporter System according to the manufacturer's instructions in a Veritas luminometer (Turner Designs). Results were expressed as changes in luciferase activity relative to vehicle-treated controls or the wild-type ABCA1 vehicle-treated control condition.

### Reverse Transcriptase PCR and Quantitative Real-time PCR

Cells were harvested for total RNA using Tri Reagent according to the manufacturer's instructions. Concentrations of total RNA were measured by spectrophotometry (Nanodrop ND-100 Spectrophotometer, Biolab). Reverse Transcriptase-PCR was performed according to the manufacturer's protocol for SuperScript III First Strand cDNA Synthesis Kit (Invitrogen). Quantitative ('real-time') Reverse Transcriptase-PCR (QRT-PCR) was performed using SensiMix dT (Quantace) on a Corbett Rotorgene 3000 and analyzed using Rotor-Gene 6 Version 6.0 (Build 27) (Corbett Research). Primer pairs used for the amplification of HMG-CoA reductase from cDNAs are F- ttggtgatgggagcttgctgtg; R- agtcacaagcacgtggaagacg. The primer pair used for amplification of the housekeeping gene PBGD was described previously [25]. PCR products were verified by sequencing. The change in gene expression levels was determined by normalizing mRNA levels of the gene of interest to the mRNA level of the house-keeping gene, porphobilinogen deaminase (PBGD). Melting curve analysis was performed to confirm production of a single product in each reaction.

### Data Presentation and Statistics

Data are presented as mean + standard error of the means (SEM) unless otherwise stated. All results are pooled or representative of at least two separate experiments. Statistical analyses were performed to find correlations (Pearson's) between two sets of continuous variables. A P-value less than 0.05 was considered statistically significant.

## Abbreviations

24,25EC, 24(S),25-epoxycholesterol; ABCA1, ATP-binding cassette transporter-A1; HMG-CoA, 3-hydroxy-3-methylglutaryl coenzyme A; LXR, liver × receptor; OSC, 2,3-oxidosqualene cyclase; SRE, sterol response element; SREBP, sterol regulatory element-binding protein; TLC, thin layer chromatography

## Supplementary Material

Additional file 1 **Validation of TLC/phosphorimaging approach for determining cholesterol and 24,25EC synthesis**. (**C**) CHO-7 cells were incubated with [1-^14^C]-acetate for 24 h under conditions of partial OSC inhibition to yield comparable levels of cholesterol and 24,25EC. Samples were pooled and neutral lipids extracted. Varying amounts of the extracts were applied to a thin-layer chromatography plate and the greatest amount was assigned an arbitrary relative concentration of 1. After development, bands corresponding to authentic cholesterol and 24,25EC were visualized by phosphorimager and quantified by densitometry (**A, B**). Alternatively, bands were cut, eluted and quantified by scintillation counting (**D, E**). Equations for the lines of best fit (**A**) y = 1.01x; R^2 ^= 0.99; (**B**) y = 1.00x; R^2 ^= 0.99; (**D**) y = 1.00x; R^2 ^= 0.99; (**E**) y = 1.00x; R^2 ^= 0.99. Click here for file

Additional file 2 **24(S),25-epoxycholesterol synthesis is increased in HepG2 cells treated with GW534511X**. HepG2 cells were incubated with increasing concentrations of GW534511X in the presence of [1-^14^C]-acetate for 24 h. Neutral lipid extracts were separated by thin-layer chromatography and bands corresponding to authentic cholesterol and 24,25EC were visualized by phosphorimager. Click here for file
