# Supplementary figures and images for: Synthesis of the oxysterol, 24(S), 25-epoxycholesterol, parallels cholesterol production and may protect against cellular accumulation of newly-synthesized cholesterol
Source: Lipids Health Dis. 2007 Apr 5;6:10. doi: 10.1186/1476-511X-6-10 (PMC1854894; doi:10.1186/1476-511X-6-10)

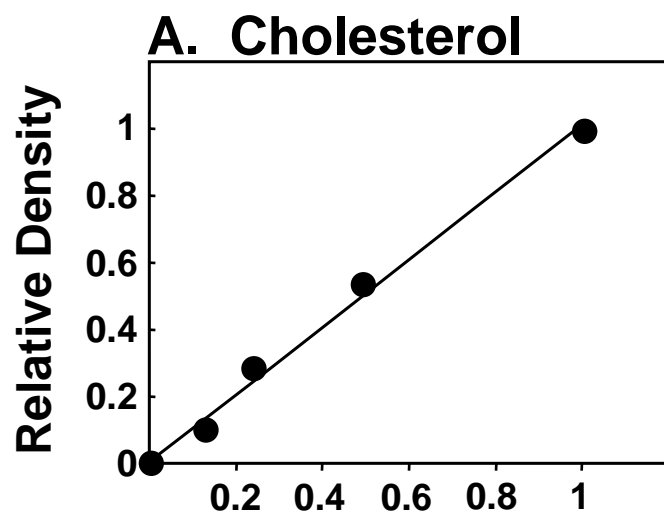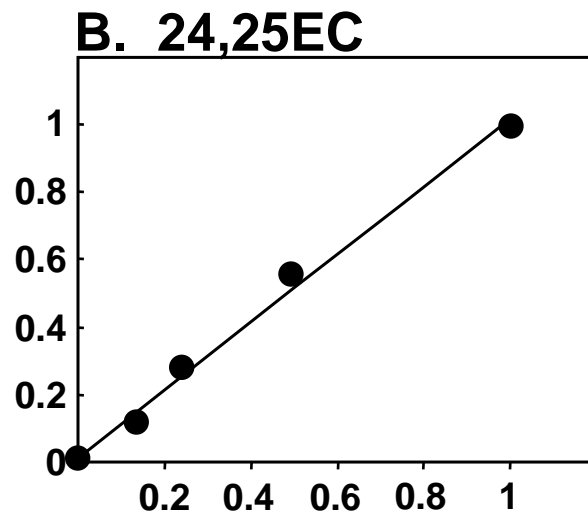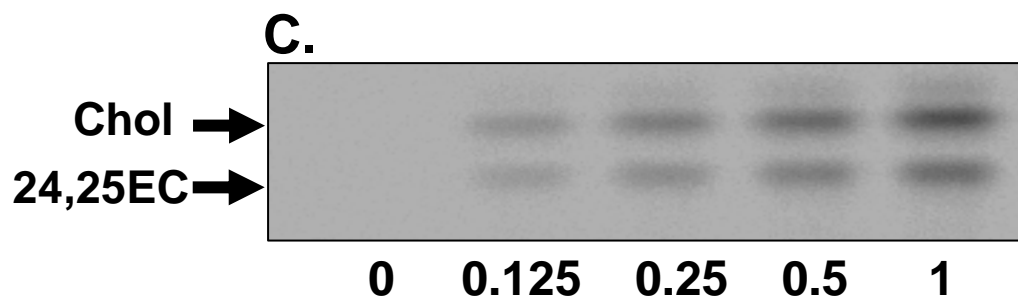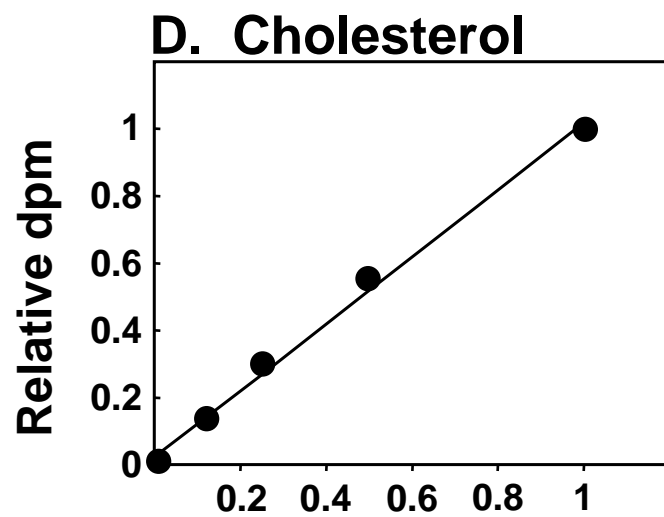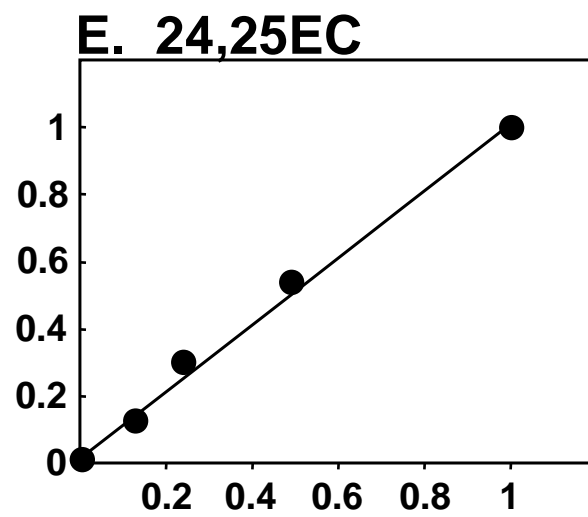

Relative Concentration

Supplement: Additional file 1 — Validation of TLC/phosphorimaging approach for determining cholesterol and 24,25EC synthesis. (C) CHO-7 cells were incubated with [1-14C]-acetate for 24 h under conditions of partial OSC inhibition to yield comparable levels of cholesterol and 24,25EC. Samples were pooled and neutral lipids extracted. Varying amounts of the extracts were applied to a thin-layer chromatography plate and the greatest amount was assigned an arbitrary relative concentration of 1. After development, bands corresponding to authentic cholesterol and 24,25EC were visualized by phosphorimager and quantified by densitometry (A, B). Alternatively, bands were cut, eluted and quantified by scintillation counting (D, E). Equations for the lines of best fit (A) y = 1.01x; R2 = 0.99; (B) y = 1.00x; R2 = 0.99; (D) y = 1.00x; R2 = 0.99; (E) y = 1.00x; R2 = 0.99. [file 1476-511X-6-10-S1.pdf]

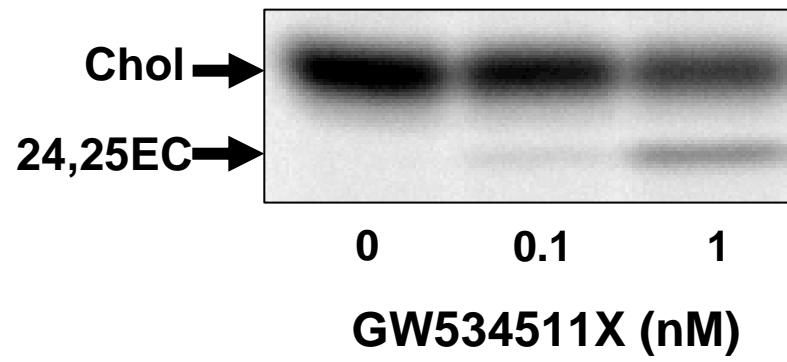

Supplement: Additional file 2 — 24(S),25-epoxycholesterol synthesis is increased in HepG2 cells treated with GW534511X. HepG2 cells were incubated with increasing concentrations of GW534511X in the presence of [1-14C]-acetate for 24 h. Neutral lipid extracts were separated by thin-layer chromatography and bands corresponding to authentic cholesterol and 24,25EC were visualized by phosphorimager. [file 1476-511X-6-10-S2.pdf]
